# Exosomes in tumor microenvironment: novel transporters and biomarkers

**DOI:** 10.1186/s12967-016-1056-9

**Published:** 2016-10-19

**Authors:** Zhen Wang, Jun-Qiang Chen, Jin-lu Liu, Lei Tian

**Affiliations:** Department of Gastrointestinal Surgery, The First Affiliated Hospital of Guangxi Medical University, 6 Shuangyong Road, Nanning, 530021 Guangxi Zhuang Autonomous Region China

**Keywords:** Tumor microenvironment, Exosomes, Transporters and biomarkers

## Abstract

Tumor microenvironment (TME) plays an integral part in the biology of cancer, participating in tumor initiation, progression, and response to therapy. Exosome is an important part of TME. Exosomes are small vesicles formed in vesicular bodies with a diameter of 30–100 nm and a classic “cup” or “dish” morphology. They can contain microRNAs, mRNAs, DNA fragments and proteins, which are shuttled from a donor cell to recipient cells. Exosomes secreted from tumor cells are called tumor-derived (TD) exosomes. There is emerging evidence that TD exosomes can construct a fertile environment to support tumor proliferation, angiogenesis, invasion and premetastatic niche preparation. TD exosomes also may facilitate tumor growth and metastasis by inhibiting immune surveillance and by increasing chemoresistance via removal of chemotherapeutic drugs. Therefore, TD-exosomes might be potential targets for therapeutic interventions via their modification or removal. For example, exosomes can serve as specific delivery vehicles to tumors of drugs, small molecules, or agents of prevention and gene therapy. Furthermore, the biomarkers detected in exosomes of biological fluids imply a potential for exosomes in the early detection and diagnosis, prediction of therapeutic efficacy, and determining prognosis of cancer. Although exosomes may serve as cancer biomarkers and aid in the treatment of cancer, we have a long way to go before we can further enhance the anti-tumor therapy of exosomes and develop exosome-based cancer diagnostic and therapeutic strategies.

## Background

Malignant tumors are complex structures that consist of cancer cells and the surrounding tumor stroma, which includes fibroblasts, immune cells, and endothelial cells. These surrounding cells are constantly releasing factors that directly or indirectly modify the tumor microenvironment (TME). The composition and characteristics of the TME are widely different [1]. TME plays an integral part in the biology of cancer, participating in tumor initiation, progression, and response to therapy. Factors released by tumor cells themselves contribute in creating an environment mostly favorable but sometimes detrimental to the tumor. [2]. Emerging evidence suggests that cellular and molecular components of TME and how they affect tumor initiation, progression, and advancement have become an imminent concern in cancer research. Moreover, events and molecules implicated in the cross talk between cancer cells and TME have emerged as attractive targets in anticancer intervention [3]. Therefore, understanding and control of TME is important for the prevention, diagnosis and treatment of malignant tumors.

Recently, studies find that exosomes may play a pivotal role among cancer cells and the cells in TME. Exosomes, firstly identified three decades ago in reticulocytes, are 30–100 nm in diameter and 1.13–1.19 g/mL in density, with a classic “cup” or “dish” morphology [4]. It is generally agreed that exosomes originate from multivesicular bodies and are released into the extracellular milieu upon the docking and fusion of multivesicular bodies with the plasma membrane [5]. To date, we experienced three breakthroughs in the cognition process of exosomes. Exosomes were initially considered as garbage bags for abandoned membrane parcels and molecular fragments, and then recognized as being closely related to the function of the immune system in the mid-1990s [6]. Secondly, researchers found that microRNAs (miRNAs), messenger RNAs (mRNA), DNA fragments and proteins could be loaded as “goods” in exosomes in the 2010s [7]. Thirdly, exosomes were found to function as “communication shuttles” between cells and transduct signals in recent years, which could re-encode genes of target cells and play a part in the development, invasion, metastasis and drug resistance of cancer [8].

In this review, we will discuss that exosomes can be served as a transporter in cancer development and progression, and can be used as a novel diagnostic and prognostic biomarker in clinics.

## Characteristics of exosomes

### Biogenesis, structure and contents of exosomes

In contrast with larger microvesicles, which are directly shed from the plasma membrane, exosomes originate from multivesicular bodies (MVB) within the endocytic system and are released into the extracellular milieu upon the docking and fusion of the MVB with the plasma membrane. The mechanism that meditates exosomal biogenesis remains elusive and may vary depending on cell types and functional contexts [4]. However, It is generally agreed that exosomes formation and release is regulated by the “endosomal sorting complexes required for transport” (ESCRT). In addition, members of the Rab GTPase (guanosine triphosphate) family (such as Rab11 and Rab27) are also important for MVB trafficking and exosome secretion [9].

As revealed by the transmission electron microscope (TEM) images, exosomes are typically cup-shaped extracellular vesicles with 30–100 nm diameter, composed by a lipid bilayer containing membrane proteins that surrounds a lumen comprising a broad range of biomolecules (such as carbohydrates, DNA and RNA). The composition of exosomes vary according to cell type and mechanism of biogesesis. To date, multiple proteins, mRNAs, and miRNAs have been identified in exosomes derived from different tissues [10]. These biomolecules packed into the lumen of exosomes mirror the content of cells from which the exosomes originated. Furthermore, exosome-carried biomolecules can be transferred to remote sites to regulate the function of distant cells and may affect the processes of receptor cells, especially by promoting interaction between various cells in the tumor microenvironment [11]. Therefore, exosomes play an important role in cancer development.

### Isolation and analysis of exosomes

To date, various methods are available for the isolation of exosomes, including ultracentrifugation, sucrose gradient ultracentrifugation combined with ultrafiltration centrifugation (SGUUC), magnetic activated cell sorting (MACS) and commercial kits. According to the requirements of the experiment, different methods can be used. Ultracentrifugation or SGUUC can be used when the sample is ample, and exosomes are abundant; otherwise, MACS and commercial kits can be used. Next, TEM can be used for shape and size analysis. Furthermore, western blot and flow cytometry (FCM) are usually used in the identification of exosomes by detecting specific tetraspanins (such as CD9, CD63 and Hsp 70). Finally, nucleic acid sequencing, western blot or ELISA can be used for exosome RNA and protein identification in the follow-up study.

### Exosomes and cancer

#### Role of exosomes in cancer growth

Exosomes secreted from tumor cells are called tumor-derived (TD) exosomes. TD exosomes may modulate the local growth of cancer via autocrine signals provided by exosomes. Autocrine effects of exosomes vary with cell types and cellular characteristics. For example, autocrine signals mediated by exosomes from the non-small cell lung cancer cell lines, glioma cells and gastric cancer cell lines increased cellular proliferation by increasing phosphorylation of Akt and extracellular signal regulated kinase, which, with other downstream molecules, are associated with cellular proliferation [12–14]. Additionally, heat shock proteins (HSP70 and HSP90) and survivin may be increased in exosomes because of cellular stresses. These proteins may inhibit apoptosis and increase cellular proliferation so they provide a strong stimulus to the microenvironment that can facilitate the growth of cancers [15, 16]. Oppositely, exosomes from human pancreatic cancer can increase the expression of Bax but decrease the expression of Bcl-2, resulting in the steering of tumor cells to the mitochondrial apoptotic pathway [17]. This suggested that TD exosomes might play potential anti-tumor role by inducing apoptosis in some cancers. Therefore, whether TD exosomes in vivo are beneficial or harmful to their own survival depends on the cell types and cellular characteristics, which needs to be elucidated further.

#### Role of exosomes in angiogenesis

The angiogenic process inherent to the cancer progression is a procedure of neovascular formation from preexisting blood vessels, which is caused by numerous interactions between regulators, mediators, and stimulatory molecules. Vascular endothelial growth factors (VEGF), fibroblast growth factor (FGF), transforming growth factor β (TGF-β), and IL-8 are some of the angiogenic factors that act on the regulation of migration and proliferation of endothelial cells, required for the stimulation of angiogenesis. Recently, the ability of TD exosomes to induce angiogenesis in various cancers and tumour microenvironments has been well documented. Katoh et al. found that exosomes released under hypoxic conditions contribute to the stimulation of angiogenesis [18]. Granulocyte macrophage colony stimulating factor (GM-CSF) induces hypoxia inducible factor 1 alpha (HIF-1α) in macrophages, and HIF-1α promotes neoangiogenesis. Hood JL found that melanoma exosomes could facilitate angiogenesis by inducing GM-CSF expression by endothelial cells in vitro and HIF-1α expression in pre-metastatic lymph nodes in vivo. This suggest a relationship between melanoma exosome induced endothelial GM-CSF and macrophage mediated angiogenesis in lymph nodes [19]. Src, which signals through focal adhesion kinase (FAK) in response to integrin activation, has been implicated in many aspects of tumor biology, including angiogenesis. DeRita et al. found that exosomes derived from the androgen receptor (AR)-positive prostate cancer cell line C4-2B, which are in rich in c-Src, could promote tumor angiogenesis [20]. Wang et al. reported that murine multiple myeloma exosomes which carried multiple angiogenesis-related proteins, enhanced angiogenesis and directly promoted endothelial cell growth by modulating several pathways such as signal transducer and activator of transcription 3 (STAT3), c-Jun N-terminal kinase, and p53. As a result, they concluded that multiple myeloma exosomes modulate the bone marrow microenvironment through enhancement of angiogenesis [21]. Further, Taraboletti et al. reported that matrix metalloproteinases (MMPs) within exosomes derived from endothelial cells were functionally active, and led to endothelial cell invasion and capillary-like structure formation [22]. Additionally, recent evidence highlights that proangiogenic RNAs (including mRNAs and miRNAs) within exosomes from cancer cells can induce angiogenesis and contribute to prompting the angiogenic switch [23]. Quantitative proteomics further revealed that proteins (such as Alix, and tetraspanins) within TD exosomes facilitated angiogenesis [24]. These suggested that exosomes have the ability to facilitate angiogenesis by inducing cell signalling between cells.

#### Role of exosomes in cancer invasion and metastasis

A central process in the metastatic cascade is cell invasion and migration, whereby disseminating cancer cells extravasate into distant sites and colonize secondary tissues and organs. Recent evidence highlights that TD exosomes may contribute to cancer invasion and metastasis by regulating stromal cells, remodeling the extracellular matrix (ECM) and stimulating angiogenesis [12]. TD-exosomes can induce changes in fibroblasts indicative of a transition to myofibroblasts. Myofibroblasts then may modulate the microenvironment (such as degradation of the ECM and increased production of pericellular hyaluronic acid) to facilitate the invasion of cancer cells [25]. Recently, exosomes derived from mesenchymal stem cells were shown to enhance the invasion and migration of human gastric cancer-27 cells via the induction of the epithelial–mesenchymal transition, which was achieved by activating the protein kinase B signaling pathway [14]. Further, studies reported that exosomes from colorectal cancer contain full length, signalling-competent epidermal growth factor receptor (EGFR) ligands. The invasive potential of colorectal cancer cells was found to be directly related to the concentration of exosomes containing the EGFR ligand, amphiregulin, suggesting that exosome-mediated ligand transfer contributes to cancer invasiveness and metastasis [26]. Additionally, exosomes derived from 21D1 were shown to be enriched with MMPs (such as MMP-1 and MMP-19) [27]. MMPs are capable of degrading ECM components, such as gelatin fibronectin and collagen, and correlate with high grade of cancer invasion [28]. These suggest that exosomes can enhance the degradation of ECM and promote the invasion of cancer cells to the matrix.

Metastasis formation is a complex process including (a) The degradation and remodeling of the ECM; (b) The formation of new blood vessels. Metastatic niche formation also requires exosomes. TD exosomes can participate actively in shaping a premetastatic niche by transporting several biologically active molecules that play important roles in the process of metastatic niche formation. Exosomal proteins with a positive effect on the invasive behavior of cancer cells, including MMPs, VEGF, and tetraspanins, can promote cell migration, cell spreading, and cable formation by regulating integrin compartmentalization, internalization, recycling, and signaling [23, 29, 30]. In addition to enhancing the invasiveness of cancer cells, TD exosomes contribute to the establishment a metastatic niche via the delivery of proteins (such as VEGF, FGF, and TGF-β) and RNAs (such as mRNAs and miRNAs) that support angiogenesis [30, 31].

#### Role of exosomes in tumor immunity

The proteins in an exosome are exactly similar to the proteins in its parallel cells and are cell-type specific. TD exosomes contain tumor-specific antigens that are expressed in the parent cells. Compared with the total cell lysates, the tumor antigens (such as carcinoembryonic antigen and mesothelin) in exosomes are more abundant. Currently, TD exosomes are being used as a source of tumor antigen to stimulate dendritic cells (DCs), leading to the transfer of tumor antigens to DCs and inducing CD8+ T cell dependent anti-tumor effects [32]. For example, miRNAs (such as miR-21 and miR-29a) transported by TD exosomes may act like ligands by binding to toll-like receptors and trigger the inflammatory response [33]. Recently, Besse et al. carried out a phase II clinical trial testing the clinical benefit of IFN (interferon)-γ-Dex (dendritic cell-derived exosomes) loaded with MHC (major histocompatibility complex) class I- and class II-restricted cancer antigens as maintenance immunotherapy after induction chemotherapy in patients bearing inoperable non-small cell lung cancer (NSCLC) without tumor progression. As a result, they found an increase in NKp30-dependent NK (natural killer) cell functions in a fraction of these NSCLC patients presenting with defective NKp30 expression. This phase II trial confirmed the capacity of Dex to boost the NK cell arm of antitumor immunity in patients with advanced NSCLC [34]. Therefore, the pro-immunogenic potential was identified as a key property of TD exosomes.

Via its normal functions, the immune system should minimize the progression and development of cancers; however, cancers were found to inhibit immune surveillance, leading to their more rapid growth, progression and dissemination [21]. Specifically, cancer cells have rapid growth after reception of exosomes isolated from matching malignant lesions [12–14]. Recently, exosomes were found to inhibit immunity by increasing the immune suppressive cells, minishing the proliferation and cytotoxicity of NK and T cells, and decreasing the number and functions of antigen presenting cells [35–37]. This implies that the decrease in immunity caused by neoplastic lesions is primarily mediated by TD exosomes [38].

Therefore, it is the balance of the activation level and abundance of the immune mediators and modulators in the tumor microenvironment that dictates if the inflammatory tumor response or anti-tumor immunity occurs. And exosomes have significant roles in this equilibrium.

#### Role of exosomes in drug resistance

Exosomes normally function in the export of waste products and less-needed molecules from cells. Recently, many studies evaluated the role of exosomes in drug resistance of cancers. Safaei et al. reported that the accumulation of cisplatin in the lysosomal compartment of human ovarian cancer cell lines is significantly reduced owing to the release of exosomes [39]. Also, the amount of cisplatin in exosomes released from cisplatin-resistant cells is 2.6 times higher than from cisplatin-sensitive cells after treatment with cisplatin. A similar phenomenon was also observed with melanosome [40]. These suggested that many anticancer drugs can be encapsulated in exosomes and effluxed outside of cells, and shedding of exosomes is closely related to drug resistance in many cancers.

In order to illuminate the mechanisms of exosomes in drug resistance, plenty of studies emerges in recent years. Lv et al reported that transport of MDR (multiple drug resistance)-1/P-gp by exosomes becomes a primary driver of docetaxel resistance in breast cancer models [41]. Au Yeung et al. found that miR21 was transferred from cancer-associated adipocytes or fibroblasts to the cancer cells, where it suppressed ovarian cancer apoptosis and conferred paclitaxel resistance by binding to its direct novel target, apoptotic protease activating factor-1 (APAF1) [42]. Further, exosomes from HER2-overexpressed breast cancer cell lines also contain full-length HER2 (human epidermal growth factor receptor-2) molecules, and can be combined with the HER2 antibody trastuzumab both in vivo and in vitro [43]. The exosome antibody interactions inhibit the anti-proliferative effects of trastuzumab by preventing it from binding to tumor cells, indicating that exosomes play a major role in the communication between cancer cells and can be used as vector-carried predictive markers and new therapeutic drugs.

The above studies support the viewpoint that the exclusion of drugs by exosomes will result in a reduction of drugs in tumors, suggesting that TD exosomes can be used as a pathway of chemoresistance of specific cancer cells to specific drugs. However, more research is needed to clarify this new mechanisms of drug efflux.

### Clinical utility of exosomes in cancer

The molecular features of TD exosomes mirror many of the molecular features of the tumors from which they originate. It was reported that TD exosomes contain biomarkers characteristic of tumors, including those of the stomach, brain, colorectum, bladder, kidney, and melanomas, so the presence of biomarkers characteristic of tumors in exosomes of biological fluids may aid in clinical decisions, including risk assessment, diagnosis, guiding treatment and determining prognosis. Of special importance is that exosomes are carried in blood and are shed into biological fluids, such as ascites and pleural fluids, all of which can be obtained easily for clinical use [44].

### Use of exosomes in cancer diagnosis

As discussed above, exosomes derived from cancer cells are enriched with proteins, mRNA, and miRNA that are more abundant in cancer cells than in normal cells [45]. Thus, exosomes may be used as biomarkers in the diagnosis of cancer. Several studies found that exosomes derived from specific tumors can be used as biomarkers for cancer diagnosis by using the method of proteomics and transcriptomics [46, 47]. For example, Vardaki et al. compared the proteomic content of exosomes derived from metastatic and non-metastatic human (MCF7 and MDA-MB-231) and mouse (67NR and 4T1) cell lines, and they identified periostin as a protein that is enriched in exosomes secreted by metastatic cells and validated its presence in a pilot cohort of breast cancer patient samples with localized disease or lymph node metastasis [48]. By profiling of human lncRNAs in prostate cancer and their exosomes from five different cell lines, Ahadi et al. identified a list of statistically significant prostate cancer lncRNAs which are differentially expressed in the exosomes compared to their parent cell lines [49]. These unique signatures make exosomes a potential tool for cancer detection.

Additionally, biological molecules characteristic of cancers can be detected in exosomes derived from various bodily fluids, such as serum, urine, saliva and malignant ascites. For example, Taylor and Gercel-Taylor observed that the levels of 8 microRNAs are of diagnostic value in ovarian cancer. They found that these microRNAs could be detected in circulating exosomes in the serum of patients, and the levels of the microRNAs found in the exosomes were similar to those found in the cancer cells, suggesting an effective way to diagnose ovarian cancer even in asymptomatic patients [50]. As an oncogene, full length EGFR has been identified in exosomes isolated from prostate cancer cells and prostate cancer patient serum, which suggested that presence of exosomal EGFR in prostate cancer patient exosomes may present a novel approach for measuring of the disease state [51]. Furthermore, Machida et al. tested the expression of four miRNAs (miR-1246, miR-3976, miR-4306, and miR-4644) in salivary exosomes from patients with pancreatobiliary tract cancer, as a result, they found that miR-1246 and miR-4644 were significantly higher in the cancer group than those in the control group. With the combination of miR-1246 and miR-4644, the area under receiver operating characteristic curve reached up to 0.833, which showed that miR-1246 and miR-4644 in salivary exosomes could be candidate biomarkers for pancreatobiliary tract cancer [52]. These evidence imply that bodily fluid derived exosomes could be a noninvasive tool for the early diagnosis of cancer. And more studies are needed to evaluate the feasibility of bodily fluid derived exosomes for cancer diagnosis.

### Use of exosomes in cancer therapy

To date, several clinical studies reported the role of exosomes in cancer therapy, and few major adverse effects were reported.

Firstly, because TD exosomes play an important role in cancer growth, metastasis and drug resistance, we can surpass this negative effect in cancer treatment by impairing the secretion of exosomes or removing exosomes from the blood circulation. Ostrowski et al. found that knockdown of Rab27 or their effectors, SYTL4 and EXPH5, could inhibit secretion of exosomes in HeLa cells [53]. GW4869S, a chemical inhibitor of sphingomyelinase 2 (which regulates the biosynthesis of ceramide and triggers the inward budding of exosomes from the limiting membrane of multivesicular endosomes), can inhibit exosome mediated tumor growth and metastasis [33]. Furthermore, Gamperl et al. found that tinzaparin (a low-molecular-weight heparin) induced tissue factor pathway inhibitor (TFPI) release from tumor cells, and recombinant TFPI inhibited tumor exosomes induced tumor cell migration [54]. Recently, to remove exosomes from the blood circulation, a therapeutic hemofiltration approach called ADAPT™ (adaptive dialysis-like affinity platform technology) was derived. As a patient’s blood passes through ADAPT™ system, plasma components through the porous fibers and interact with the immobilized affinity agents to which target molecules are selectively adsorbed while blood cells and unbound serum components pass through the system. Thus, the capability for removing circulating exosomes of ADAPT™ could offer a unique strategy for improving the therapeutic outcomes for cancer patients [55]. However, since exosome production is determined by the size and growth rate of the tumor [55], the duration and frequency of ADAPT™ therapy would require optimizing to achieve a clinically beneficial level of circulating exosome depletion. The device strategy could also be tailored for different types/stages of cancer and using devices incorporating different affinity agents. Currently, although a spectrum of biologic effects of cancer exosomes have been identified in vitro and experimental animals, the impact of clearing exosomes therapeutically must still be evaluated the potential efficacy in promoting immune recovery and hindering tumor growth in a clinical setting.

Secondly, exosomes can be used as delivery vehicles loaded with various anticancer drugs, miRNAs, and siRNAs. In principle, exosomes used as drug delivery vehicles have multiple advantages over existing synthetic systems: (1) As exosomes can be derived from autologous tumor cells, they may be less immunogenic than artificial delivery vehicles, thus probably leading to minimal toxicities when being transferred into target cells. (2) Exosomes have phospholipid bilayers, which may directly fuse with the target cell plasma membrane, thus improve the cellular internalization of the encapsulated drug. (3) The naturally small size of exosomes allows them to avoid phagocytosis by the mononuclear phagocyte system and facilitates their extravasation through tumor vessels and their subsequent diffusion in tumor tissues. Several studies have described the successful delivery and tumor inhibition effect of TD exosomes. For example, exosome-delivered tumor suppressor miRNAs, miR-143, and let-7a, inhibited the growth of prostate and breast cancer in vivo, respectively. No adverse effects were observed in normal prostatic epithelial cells after treatment with exosome-encapsulated miR-143 [56, 57]. Also, doxorubicin loaded into exosomes or exosome-mimetic nanovesicles inhibited the growth of colon and breast cancer xenograft tumors in vivo [58, 59]. Furthermore, by targeting immature dendritic cell exosomes directly to the tumor tissue, the efficacy of doxorubicin was greatly enhanced. The enhanced efficacy was accompanied with significantly less adverse effects on the main organ systems, implying that delivery via exosomes might decrease the major downside of this chemotherapeutic drug [58]. These results suggest an opportunity for using exosomes as a carrier of treatments of cancer. Considering exosomes’ rate of clearance and biodistribution, administration routes used for exosome drug delivery should be studied in future.

Thirdly, because exosomes have a specific cell tropism according to their characteristics, they can be used to target specific tissues or organs. For example, two studies bioengineered a well-characterized exosomal membrane protein (Lamp2b) to express a targeting peptide immediately below the signal peptide sequence, which secured the correct insertion of the protein into the exosome membrane and avoided cleavage of important regions in the targeting peptide sequence. Using this method, the targeting peptides RVG and iRGD were successfully inserted into exosomes from immature dendritic cells to target either brain or tumor tissues [58, 60]. Both studies found that usage of a targeting peptide on a Lamp2b pedestal significantly increased the specificity of the treatment, enhanced the cellular uptake of exosomes in the tissues of interest, and decreased the toxicity of drugs delivered by exosomes. These results suggest that exosomes modified by targeting ligands can be used therapeutically for the delivery of an anti-cancer substance to tumors, thus having great potential value for clinical applications.

At last, because of the role of exosomes in the immune system, exosome based cell free vaccines could represent an alternative to dendritic cell (DC) therapy for suppressing tumor growth. By transfecting B16 melanoma cells with a plasmid encoding the Mycobacterium tuberculosis antigen, Koyama et al. collected the secreted exosomes bearing the pathogenic antigen, and then injected the exosomes into foot pads of mice. As a result, they found these exosomes significantly (p < 0.05) evoked cellular immunity against B16 tumor cells, and suppressed (p < 0.001) tumor growth in syngeneic B16 tumor-bearing mice [61]. This suggests that exosomes bearing both tumor antigens and the Mycobacterium tuberculosis antigen have a high potential as a candidate for cancer vaccine to overcome the immune escape by tumor cells. Similarly, by loading Dex with IFN-γ, MHC class I- and class II-restricted cancer antigens, Besse et al. carried out a phase II clinical trial to test these exosomes as maintenance immunotherapy after induction chemotherapy in patients bearing inoperable NSCLC. And at last they confirmed the capacity of Dex to boost the NK cell arm of antitumor immunity in patients with advanced NSCLC [34]. Although these findings are encouraging, some researchers observed that only a small percentage of patients presented transient stabilisation of the disease [62]. This implies that more studies are needed to evaluate the role of exosomes in immune therapy of cancer.

### Use of exosomes as prognostic biomarkers for cancers

Each tumor is characterized by a specific miRNA or protein profile. Increasing evidence has shown that exosomal miRNAs and proteins are positively correlated with the stage and degree of tumor progression. Vered et al. investigated caveolin-1 (CAV1) in exosomes of tongue squamous cell carcinoma (TSCC) and its association with clinical outcomes. As a result, they found that expression of CAV1 in TSCC had a higher score in exosomes than in the tumor cells and a negative impact on recurrence (p = 0.01) and survival (p = 0.003), which suggested that accumulation of CAV1 in exosomes had a negative prognostic value in TSCC [63]. In addition, Ye et al. isolated exosomes from serum of healthy donors and patients with nasopharyngeal carcinoma (NPC). They found that miR-24-3p was markedly enriched in exosomes from patients with NPC compared with those from healthy donors; and the serum exosomal miR-24-3p level was correlated with worse disease-free survival of patients (p < 0.05) [37]. These allow the use of TD exosomes as novel prognostic markers.

## Summary

Exosomes, as small particles, can function as key transporters for intercellular communication and for modulating immune responses, owing to their content of proteins and genetic material that mirror their cells of origin. Tumors have hijacked this mechanism of intercellular communication to aid in their growth, progression, and dissemination [64]. TD-exosomes can construct a fertile environment to support tumor proliferation, angiogenesis, invasion and premetastatic niche preparation. TD-exosomes also may facilitate tumor growth and metastasis by inhibiting immune surveillance and by increasing chemoresistance via removal of chemotherapeutic drugs (Table 1). Therefore, TD-exosomes might be potential targets for therapeutic interventions via their modification or removal. For example, exosomes can serve as specific delivery vehicles to tumors of drugs, small molecules, or agents of prevention and gene therapy. Furthermore, the biomarkers detected in exosomes of biological fluids imply a potential for exosomes in the early detection and diagnosis, prediction of therapeutic efficacy, and determining the prognosis of cancer (Table 2). However, many problems still need to be resolved, such as (1) The use of exosomes as cancer biomarkers is technically limited by their size, heterogeneity and the need for extensive purification and labelling; (2) How to use exosomes as delivery vehicles loaded with anticancer drugs effectively in clinics? and (3) How to block the negative effects of TD exosomes on tumors? Therefore, we have a long way to go before we can further enhance the anti-tumor therapy of exosomes and develop exosome-based cancer diagnostic and therapeutic strategies.

**Table 1:** The relationships between exosomes and cancer

Study ID (author, year)	Study design	Main results
Gu et al., 2016 [14]	The effects of MSC-exosomes on gastric cancer are evaluated, and the possible mechanisms are explored	Exosomes derived from human mesenchymal stem cells promote gastric cancer cell growth and migration by activating the Akt pathway
DeRita et al., 2016 [20]	The effects of exosomes derived from prostate cancer cell on tumors and the contents of these exosomes are evaluated	Src, IGF-IR, and FAK are enriched in exosomes from the AR-positive cell line C4-2B, which are implicated in many aspects of tumor biology including angiogenesis
Wang et al., 2016 [21]	The functions of MM exosomes in angiogenesis and immunosuppression are explored in vitro and in vivo	Murine MM exosomes which carried multiple angiogenesis-related proteins, enhanced angiogenesis and directly promoted endothelial cell growth by modulating several pathways such as STAT3
Khalyfa et al., 2016 [12]	The effects of exosomes from OSA patients on human adenocarcinoma cells are evaluated	OSA induces alterations in exosomal miRNA cargo that alter the biological properties of TC1 lung tumor cells to enhance their proliferative, migratory and extravasation properties
Fabbri et al., 2012 [33]	The expression level of miR-21 and miR-29a in exosomes derived from lung cancer cell lines and their effects on tumor immune are evaluated	MiR-21 and miR-29a are highly expressed in exosomes derived from lung cancer cell lines, function by binding as ligands to receptors of TLR family (murine TLR7 and human TLR8) in immune cells, and trigger a TLR-mediated prometastatic inflammatory response that ultimately may lead to tumor growth and metastasis
Au Yeung et al., 2016 [42]	The level of miRNA21 in exosomes isolated from cancer-associated adipocytes and fibroblasts and its effects on ovarian cancer cells are evaluated	The level of miRNA21 in exosomes isolated from cancer-associated adipocytes and fibroblasts is significantly higher than in those from ovarian cancer cells. Functional studies reveal that miR21 is transferred from cancer-associated adipocytes or fibroblasts to the cancer cells, where it suppresses ovarian cancer apoptosis and confers chemoresistance by binding to APAF1

*MSC* mesenchymal stem cell, *IGF-IR* insulin like growth factor-I receptor, *FAK* focal adhesion kinase, *AR* androgen receptor, *MM* multiple myeloma, *STAT3* signal transducer and activator of transcription 3, *OSA* obstructive sleep apnea, *TLR* toll-like receptor, *APAF1* apoptotic protease activating factor-1

**Table 2:** Clinical utility of exosomes in cancer

Study ID (author, year)	Study design	Main results
Kharmate et al., 2016 [51]	The expression level of EGFR in exosomes derived from prostate cancer cells and prostate cancer patient serum	Presence of exosomal EGFR in prostate cancer patient exosomes may present a novel biomarker for measuring of the disease state
Machida et al., 2016 [52]	The expression of four miRNAs (miR-1246, miR-3976, miR-4306, and miR-4644) in salivary exosomes from patients with pancreatobiliary tract cancer	MiR-1246 and miR-4644 were significantly higher expressed in the cancer group than those in the control group, suggesting that miRNAs in exosomes can be used as candidate biomarkers for tumors
Tian et al., 2014 [58]	Exosomes derived from mouse immature dendritic cells are loaded with doxorubicin via electroporation	Intravenously injected exosomes deliver doxorubicin specifically to tumor tissues and lead to inhibition of tumor growth without overt toxicity, suggesting that exosomes can be used as transporters loaded with various anticancer drugs
Koyama et al., 2016 [61]	Exosomes derived from B16 melanoma cells which are transfected with a plasmid encoding the Mycobacterium tuberculosis antigen are collected and injected into foot pads of mice	The modified exosomes significantly evoked cellular immunity against B16 tumor cells, and suppressed tumor growth in syngeneic B16 tumor-bearing mice, suggesting that exosomes have a high potential as a candidate for cancer vaccine to overcome the immune escape by tumor cells
Vered et al., 2015 [63]	They investigate CAV1 in exosomes of TSCC and its association with clinical outcomes	The expression of CAV1 in TSCC had a higher score in exosomes than in the tumor cells and a negative impact on recurrence and survival, suggesting that exosomes can be used as novel prognostic biomarkers for tumors

*EGFR* epidermal growth factor receptor, *CAV1* Caveolin-1, *TSCC* tongue squamous cell carcinoma

## Abbreviations

ADAPT™: adaptive dialysis-like affinity platform technology
APAF1: apoptotic protease activating factor-1
AR: androgen receptor
CAV1: caveolin-1
DC: dendritic cells
Dex: dendritic cell-derived exosomes
ECM: extracellular matrix
EGFR: epidermal growth factor receptor
ESCRT: endosomal sorting complexes required for transport
FAK: focal adhesion kinase
FCM: flow cytometry
FGF: fibroblast growth factor
GM-CSF: granulocyte macrophage colony stimulating factor
GTP: guanosine triphosphate
HER2: human epidermal growth factor receptor-2
HIF-1α: hypoxia inducible factor 1 alpha
IFN: interferon
HSP: heat shock proteins
MACS: magnetic activated cell sorting
MDR: multiple drug resistance
MHC: major histocompatibility complex
MMP: matrix metalloproteinase
MVB: multivesicular bodies
NK: natural killer
NPC: nasopharyngeal carcinoma
NSCLC: non-small cell lung cancer
PSA: prostate-specific antigen
PSMA: prostate-specific membrane antigen
SGUUC: sucrose gradient ultracentrifugation combined with ultrafiltration centrifugation
STAT3: signal transducer and activator of transcription 3
TD exosomes: tumor-derived exosomes
TEM: transmission electron microscope
TFPI: tissue factor pathway inhibitor
TGF: transforming growth factor
TME: tumor microenvironment
TSCC: tongue squamous cell carcinoma
VEGF: vascular endothelial growth factors
